# Supplementary material for: Single-molecule FRET unmasks structural subpopulations and crucial molecular events during FUS low-complexity domain phase separation
Source: Nat Commun. 2023 Nov 13;14:7331. doi: 10.1038/s41467-023-43225-y (PMC10643395; doi:10.1038/s41467-023-43225-y)
Supplement: Supplementary file 1 — Supplementary Information [file 41467_2023_43225_MOESM1_ESM.pdf]

Supplementary Information

Single-molecule FRET unmasks structural subpopulations and crucial molecular events during FUS low-complexity domain phase separation

Ashish Joshi,[1,2] Anuja Walimbe,[1,2] Anamika Avni,[1,3] Sandeep K. Rai,[1,3] Lisha Arora,[1,3] Snehasis Sarkar,[1,2] and Samrat Mukhopadhyay[1,2,3*]

[1]Centre for Protein Science, Design and Engineering, [2]Department of Biological Sciences, and [3]Department of Chemical Sciences, Indian Institute of Science Education and Research (IISER) Mohali, Punjab, India. *Corresponding author: mukhopadhyay@iisermohali.ac.in

Table of Contents **Page number**

1. Materials.. S2
2. Supplementary Tables.. S3
3. Supplementary Figures.. S7
4. Supplementary References.. S13

Supplementary Materials

The catalog number for all the materials are mentioned in parentheses. Sodium phosphate monobasic dihydrate (P9791), sodium phosphate dibasic dihydrate (71643), 2-mercaptoethanol (BME) (M3148), 1,4-dithiothreitol (DTT) (43815), Tris(2-carboxyethyl)phosphine hydrochloride (TCEP) (C4706), and Urea (U5378) were of MB grade purity, procured from Sigma (St. Louis, MO, USA). Luria Bertani Broth, Miller (LB) (M1245), N-cyclohexyl-3-aminopropanesulfonic acid (CAPS) (MB008), sodium chloride (1.93206.0521), ethylenediaminetetraacetic acid (EDTA) (MB011), and nickel chloride (GRM1394) were procured from HiMedia Laboratories. Kanamycin (K-120-10) and isopropyl-β-thiogalactopyranoside (IPTG) (I2481) were obtained from Gold Biocom (USA). Fluorescent probes like fluorescein-5-maleimide (F-5-M) (F150), AlexaFluor488 succinimidyl ester, AlexaFluor488 (A20000), and AlexaFluor594-maleimide (A10256) were purchased from Molecular Probes, Invitrogen. Ni-NTA resin (30230) was purchased from Qiagen. Amicon membrane filters (UFC901024) for concentrating protein were obtained from Merck Millipore. PD-10 (17085101), NAP-10 (17085402), and HiLoad 16/600 Superdex-G-200 (28-9893-23) columns were purchased from GE Healthcare Life Sciences (USA). High-purity milli-Q water was used to prepare all the buffers in this study. A Metrohm 827 lab pH meter was used to adjust the pH (± 0.01) of all the buffer solutions prepared at 25 °C, and all the buffer solutions were filtered before use.

Supplementary Tables

Supplementary Table 1. Primers used for site-directed mutagenesis.

A16C Forward	CCCAAAGCTATGGGTGCTACCCCACCCAGC
A16C Reverse	GCTGGGTGGGGTAGCACCCATAGCTTTGGG
S86C Forward	CTATGGCAGTAGCCAGTGCTCCCAATCGTC
S86C Reverse	GACGATTGGGAGCACTGGCTACTGCCATAG
S108C Forward	CCAGCTCCCAGCTGCACCTCGGGAA
S108C Reverse	TTCCCGAGGTGCAGCTGGGAGCTGG
S148C Forward	AAAGCTATGGACAGCAGCAATGCTATAATCCCCC
S148C Reverse	GGGGGATTATAGCATTGCTGCTGTCCATAGCTTT
LC G156E Forward	GCTATAATCCCCCTCAGGGCTATGAACAGCAGAACCAGTACAACAGC
LC G156E Reverse	GCTGTTGTACTGGTTCTGCTGTTCATAGCCCTGAGGGGGATTATAGC

Supplementary Table 2. Observed FRET efficiencies varying the inter-residue length estimated from single-molecule FRET data analyses and the comparison with the calculated FRET efficiencies based on the random coil model.[1] The approximate inter-dye distances estimated from FRET peaks of single-molecule FRET histograms are shown in parenthesis.

Constructs	Number of residues	Calculated FRET efficiencies	Experimental FRET efficiencies (Inter-dye distance in Å)
N-to-86	86	0.32	0.76 ± 0.02 (44.6 Å)
N-to-108	108	0.16	Subpopulation 1: 0.80 ± 0.02 (42.9 Å)<br>Subpopulation 2: 0.97 ± 0.03 (30.3 Å)
N-to-148	148	0.06	Subpopulation 1: 0.73 ± 0.01 (45.8 Å)<br>Subpopulation 2: 0.09 ± 0.02 (79.4 Å)

Supplementary Table 3. FRET efficiencies varying the inter-residue length estimated from single-molecule FRET data analyses for wild-type and G156E FUS-LC condensates.

Constructs	FRET efficiencies from single-droplet single-molecule FRET studies for wild-type FUS-LC	FRET efficiencies from single-droplet single-molecule FRET studies for G156E FUS-LC
N-to-86	0.64 ± 0.01	0.64 ± 0.01
N-to-108	Subpopulation 1: 0.79 ± 0.08<br>Subpopulation 2: 1.00 ± 0.01	Subpopulation 1: 0.86 ± 0.04<br>Subpopulation 2: 1.01 ± 0.01
N-to-148	Subpopulation 1: 0.30 ± 0.01<br>Subpopulation 2: 0.90 ± 0.02	Subpopulation 1: 0.24 ± 0.02<br>Subpopulation 1: 0.79 ± 0.06

Supplementary Table 4. Rotational correlation times and associated amplitudes recovered by fitting fluorescence anisotropy decay kinetics using a biexponential decay model for the monomeric dispersed phase and individual droplets.

**Residue position**		**Fast rotational correlation time (ϕ_1) and amplitude (β_1)**	**Slow rotational correlation time (ϕ_2) and amplitude (β_2)**
**16**	Monomer	0.95 ± 0.12 ns (0.66 ± 0.08)	4.34 ± 0.45 ns (0.33 ± 0.08)
	Droplet	1.02 ± 0.16 ns (0.29 ± 0.12)	57.73 ± 5.44 ns (0.74 ± 0.01)
**86**	Monomer	0.77 ± 0.07 ns (0.66 ± 0.05)	4.11 ± 0.32 ns (0.33 ± 0.05)
	Droplet	1.42 ± 0.09 ns (0.26 ± 0.00)	62.49 ± 4.29 ns (0.73 ± 0.00)
**108**	Monomer	0.93 ± 0.06 ns (0.65 ± 0.02)	5.61 ± 0.81 ns (0.34 ± 0.02)
	Droplet	1.11 ± 0.08 ns (0.23 ± 0.01)	58.26 ± 1.37 ns (0.76 ± 0.01)
**148**	Monomer	0.92 ± 0.06 ns (0.79 ± 0.05)	5.16 ± 0.70 ns (0.20 ± 0.05)
	Droplet	1.29 ± 0.24 ns (0.21 ± 0.02)	63.50 ± 3.92 ns (0.78 ± 0.02)

Supplementary Table 5. Raman shift values and tentative band assignments of single-droplets of LC WT and LC G156E based on previous protein Raman studies.[2-7]

LC WT (cm^{-1})	LC G156E (cm^{-1})	Peak Assignment*
645	644	Tyr [γ(C-C)]
831	829	Tyr Fermi Doublet
855	853	Tyr Fermi Doublet
992	990	Dibasic Phosphate (buffer)
1046	1042	Proline CH_2 wagging
1180	1178	Tyr, ν(C-N)
1212	1210	Tyr [ν(C-C)]
1255	1256	Amide III
1338	1341	δ(CαH)
1424	1424	ν(C-H)
1455	1453	δ(CH_2/CH_3)
1620	1619	Tyr (R stretch)
1671	1676	Amide I

*δ, bending; ν, stretching; γ, twisting.

Supplementary Figures

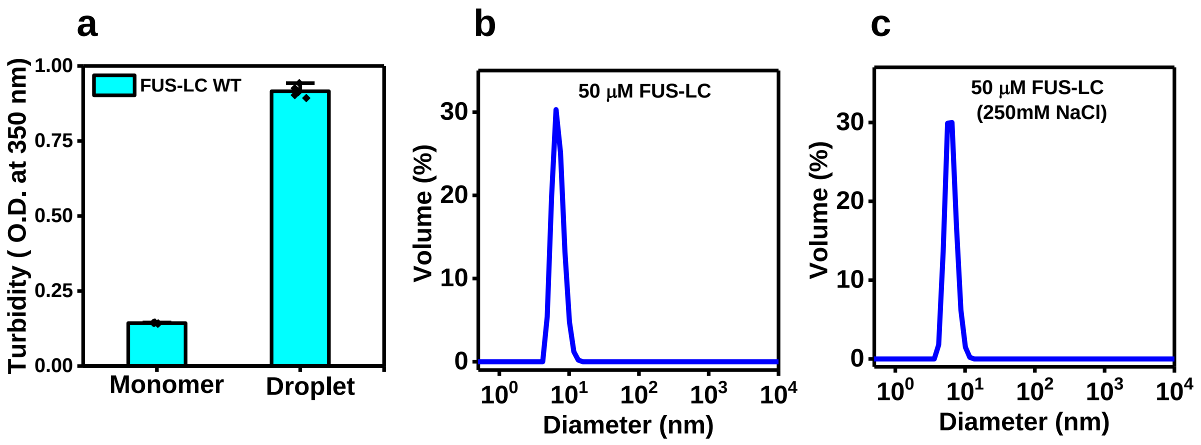

Supplementary Figure 1. a. Turbidity plot of wild-type FUS-LC measured at 350 nm in monomeric and droplet conditions. Turbidity measurements were performed for 200 µM of FUS-LC in 20 mM phosphate buffer, pH 7.4 without salt and with 250 mM NaCl for monomer and droplet phases, respectively. Data represent mean ± SD (n = 5 independent reactions). Representative distribution of particle size obtained from dynamic light scattering (DLS) measurements of monomeric FUS-LC (50 µM protein, in 20 mM phosphate, pH 7.4) under non-phase separating (no salt) (b) and phase-separating conditions (c) (250 mM NaCl). The experiment was independently repeated 3 times with similar results.

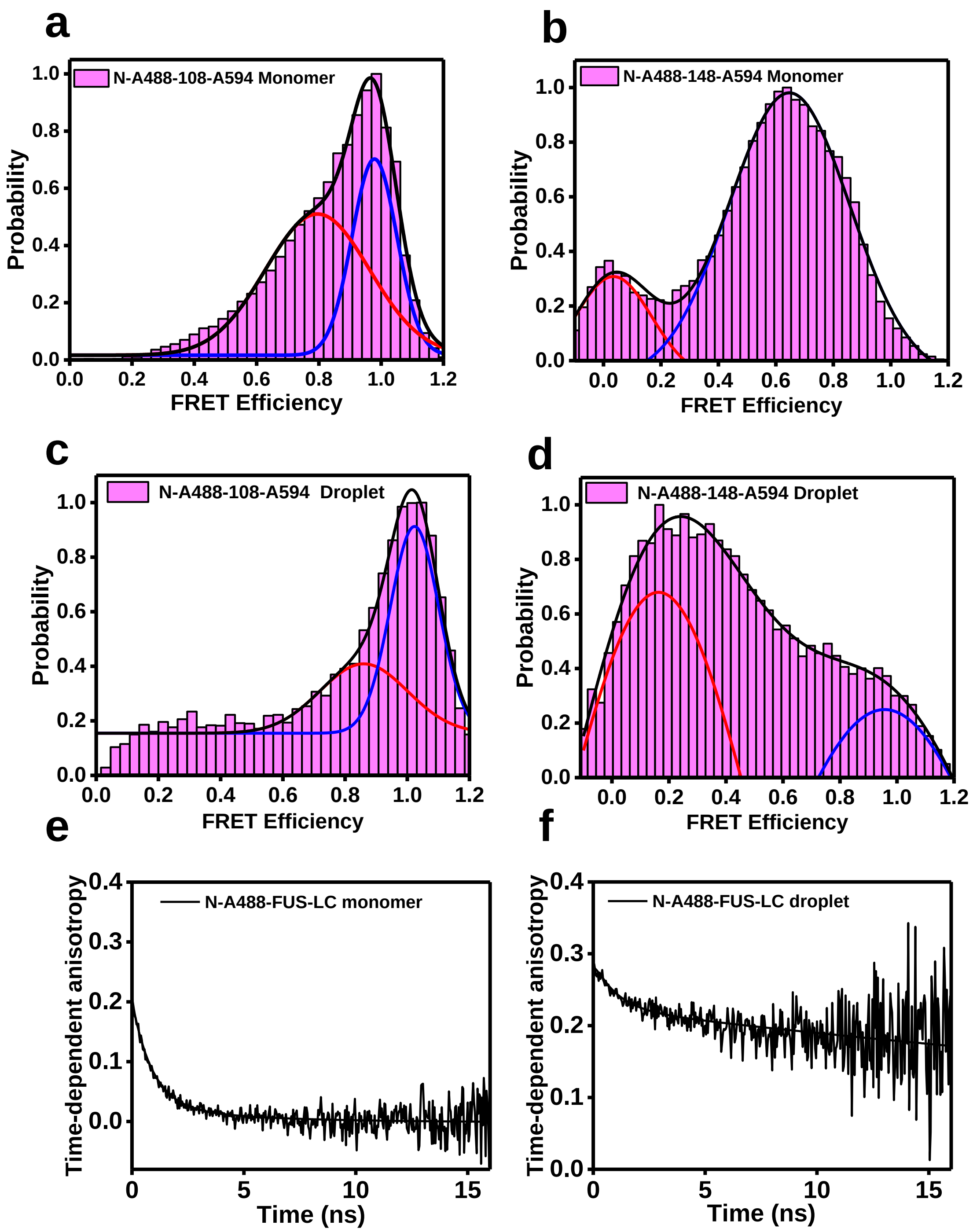

Supplementary Figure 2. The effect of binning time showing no significant changes in the FRET efficiency histograms. These results with a 1 ms binding time are similar to the results with 0.5 ms binning time shown in Figure 3. Single-molecule FRET efficiency histograms obtained for the dispersed phase of N-to-108 (a) and N-to-148 (b) constructs acquired in the presence of 75-150 pM dual-labeled FUS-LC. Single-droplet single-molecule FRET efficiency histograms obtained for the condensed phase of N-to-108 (c) and N-to-148 (d) constructs acquired in the presence of 5-10 pM dual-labeled FUS-LC. All the single-molecule FRET

measurements were performed in 20 mM phosphate buffer, 250 mM NaCl, pH 7.4, the donor and acceptor emissions were recorded in the PIE mode and FRET efficiency distribution was obtained with a binning time of 1 ms using SymphoTime64 software v2.7. Representative picosecond time-resolved fluorescence anisotropy decay profiles for FUS-LC N-terminally labeled with AlexaFluor488 (donor) within the monomeric dispersed (e) and droplet (f) phases. Solid lines represent fits obtained from biexponential decay analysis. Fast rotational correlation times and associated fractional amplitudes obtained within the monomeric phase (~ 0.9 ns and 0.86) and condensed phase (~ 1.8 ns and 0.22) indicated considerable depolarization due to local rotational dynamics of the attached fluorophore. Similar parameters were recovered for the acceptor (AlexaFluor594)-labeled FUS-LC.

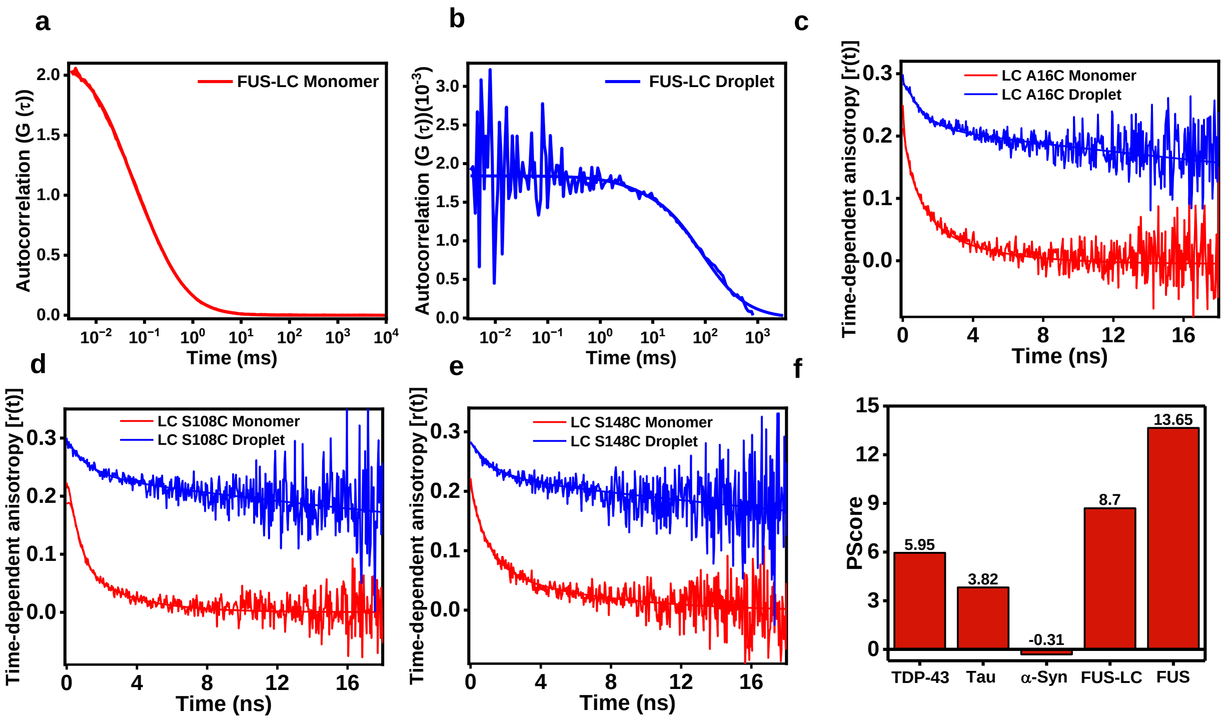

Supplementary Figure 3. Unnormalized representative FCS autocorrelation plots with fits for the monomeric (a) and condensed phase (b) of wild-type FUS-LC in the presence of 10 nM (monomer) and 1-3 nM (droplet) AlexaFluor488-labeled FUS-LC. The normalized FCS plots are shown in Fig. 4a. c-e. Representative time-resolved fluorescence anisotropy decay of single-cysteine variants of FUS-LC obtained within the dispersed phase and single droplets (F-5-M labeled at residue locations 16, 108, and 148) (c), (d), and (e), respectively. Solid lines are fits obtained from biexponential decay analysis. f. Comparison of Pscore values of FUS-LC with other well-studied phase-separating proteins.

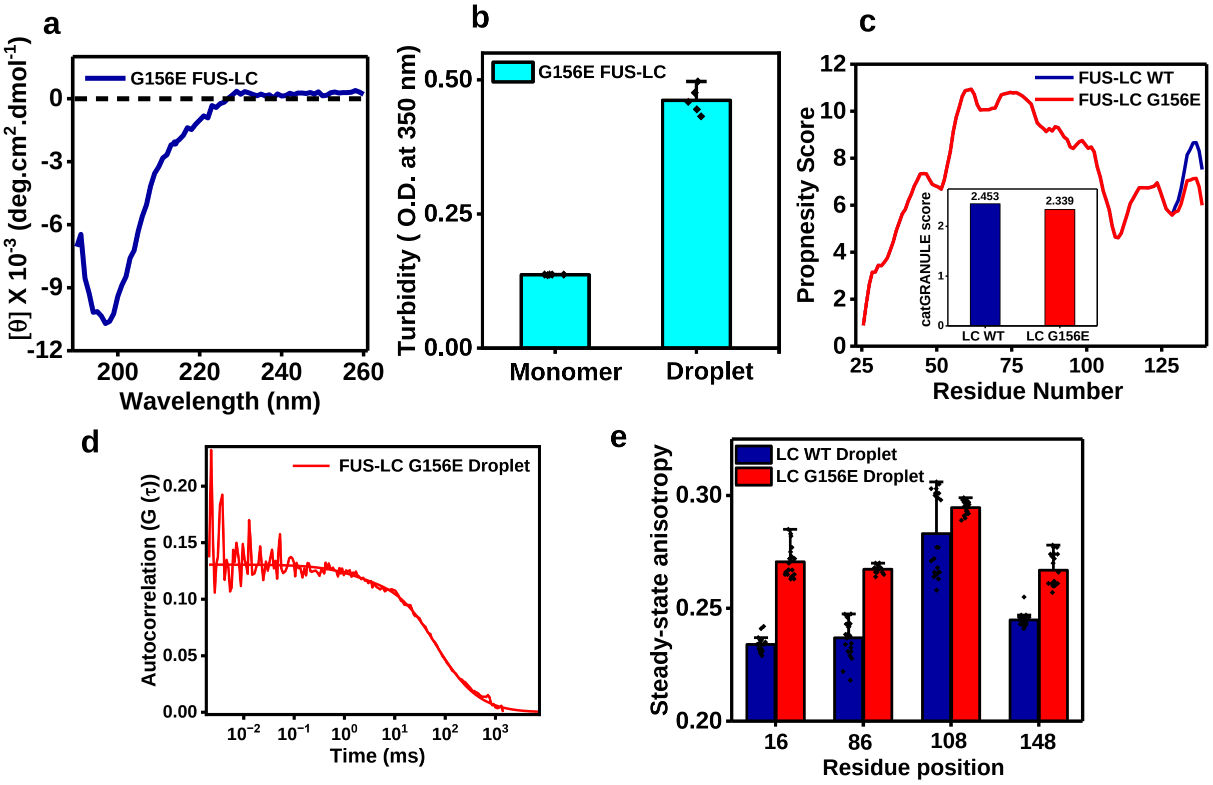

Supplementary Figure 4. a. The circular dichroism spectrum of G156E FUS-LC shows similar structural features to wild-type FUS-LC. CD measurements were performed for 10 µM G156E FUS-LC in 20 mM phosphate, pH 7.4. b. The solution turbidity plot of G156E FUS-LC (200 µM protein in 20 mM phosphate, pH 7.4) indicates phase separation in the presence of 250 mM salt. Data represent mean ± SD (n = 5 independent samples). c. Comparison of phase separation propensity plots of wild-type and mutant G156E FUS-LC using bioinformatics tool catGranule shows a slightly lower propensity of G156E FUS-LC. Inset shows a comparison of catGranule score for wild-type and G156E FUS-LC. d. Representative autocorrelation plot obtained by FCS measurements within individual condensates of G156E FUS-LC. Droplets of G156E FUS-LC were doped with 1-3 nM AlexaFluor488-labeled FUS-LC for FCS measurements. e. Comparison of single-droplet steady-state fluorescence anisotropy values within wild-type and G156E FUS-LC droplets. Steady-state fluorescence anisotropy at all the positions showed a slight increase within G156E droplets. Data represent mean ± SD for n = 20 droplets (data for wild-type FUS-LC droplets are the same as shown in Fig. 4d and included here for comparison).

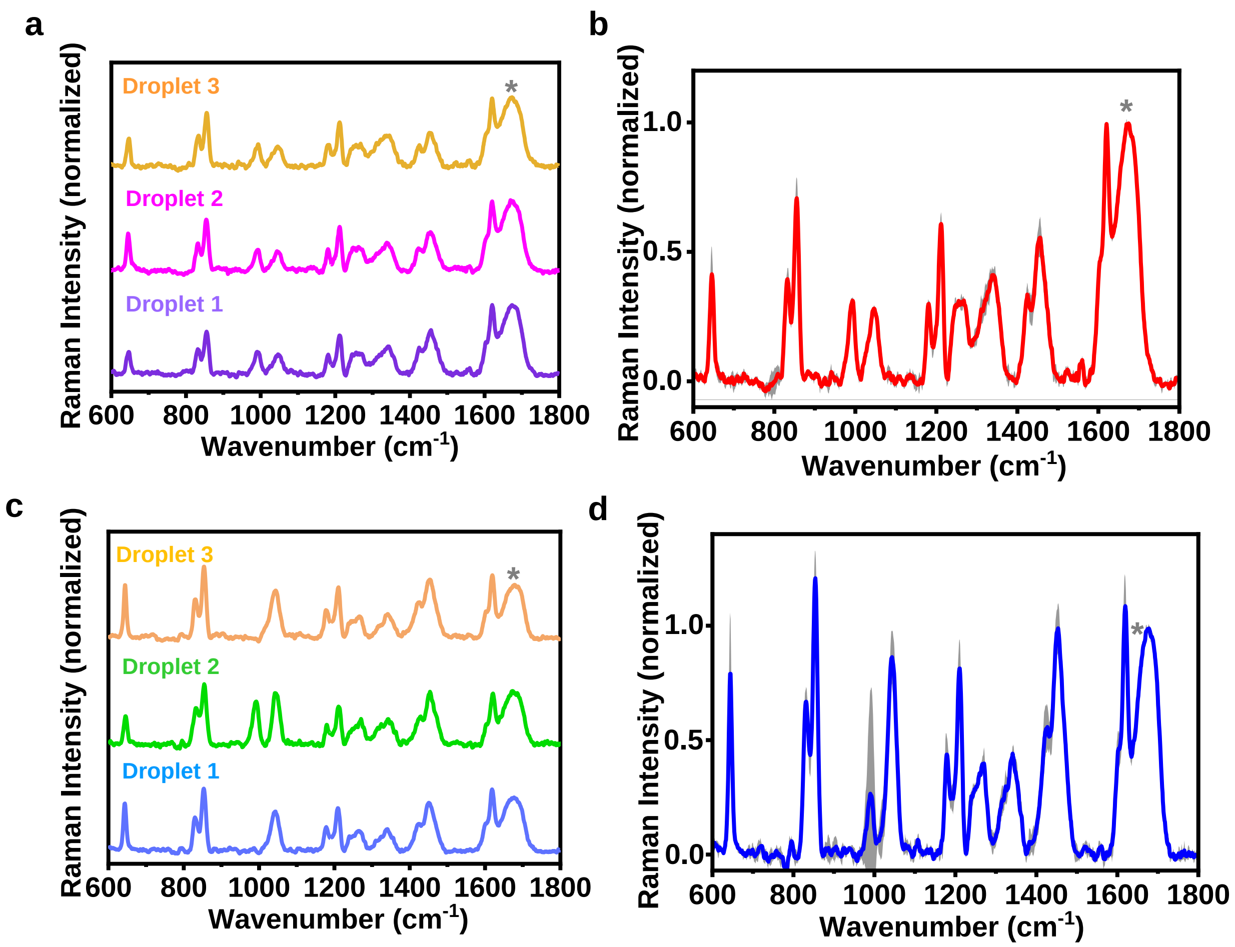

Supplementary Figure 5. a. Representative single-droplet normal Raman spectra of individual FUS-LC droplets (spectra recorded at 500 mW laser power, 100x objective; the number of droplets, n = 3). b. Mean and standard deviation of Raman spectra shown in (a). Raman spectra are normalized with respect to the amide I band at ~ 1673 cm^{-1}, marked by an asterisk. See "Methods" for details of data acquisition, processing, and analysis. c. Representative single droplet normal Raman spectra of individual G156E FUS-LC droplets (spectra recorded at 500 mW laser power, 100x objective; the number of droplets, n = 3). d. The mean and standard deviation of Raman spectra shown in (c). Raman spectra are normalized with respect to the amide I band at ~ 1673 cm^{-1}, marked by an asterisk.

Supplementary References

1. Melo, A. M., Coraor, J., Alpha-Cobb, G., Elbaum-Garfinkle, S., Nath, A., & Rhoades, E. A. Functional role for intrinsic disorder in the tau-tubulin complex. *Proc. Natl. Acad. Sci. U.S.A.* **13,** 14336-14341 (2016).
2. Rygula, A., Majzner, K., Marzec, K. M., Kaczor, A., Pilarczyk, M., Baranska, M. Raman Spectroscopy of Proteins: A Review. *J. Raman Spectrosc.* **44,** 1061-1076 (2013).
3. Tuma, R. Raman Spectroscopy of Proteins: From Peptides to Large Assemblies. *J. Raman Spectrosc.* **36,** 307-319 (2005).
4. Szekeres, G. P., Kneipp, J. Probing of Proteins in Gold Nanoparticle Agglomerates. *Front. Chem.* **7,** 30, (2019).
5. Zhu, G., Zhu, X., Fan, Q., and Wan, X. Raman spectra of amino acids and their aqueous solutions. *Spectrochim. Acta. A Mol. Biomol. Spectrosc*. **78**(3), 1187-1195 (2011).
6. Rippon, W. B., Koenig, J. L., Walton, A. G. Raman spectroscopy of proline oligomers and poly-L-proline. *J. Am. Chem. Soc.* **92**(25), 7455-7459 (1970).
7. Niaura, G., Gaigalas, A. K., and Vilker, V. L. Surface-Enhanced Raman Spectroscopy of Phosphate Anions: Adsorption on Silver, Gold, and Copper Electrodes. *J. Phys. Chem. B* 1997, **101**, 45, 9250–9262 (1997).
